# Time to interval cholecystectomy and associated outcomes in a population aged 50 and above with mild gallstone pancreatitis

**DOI:** 10.1007/s00423-023-03098-7

**Published:** 2023-09-28

**Authors:** Jian D. Blundell, Robert C. Gandy, Jacqueline C. T. Close, Lara A. Harvey

**Affiliations:** 1 Prince of Wales Hospital, Sydney, NSW Australia; 2 Neuroscience Research Australia, Sydney, NSW Australia; 3 University of NSW, Sydney, NSW Australia

**Keywords:** Gallstone pancreatitis, Interval cholecystectomy, Mortality, Emergency readmission, Length of stay, Open cholecystectomy, Endoscopic retrograde Cholangiopancreatography (ERCP)

## Abstract

**Background:**

Cholecystectomy on index admission for mild gallstone pancreatitis (GSP) is recommended, although not always feasible. This study examined rates and outcomes of people aged ≥ 50 years who underwent interval (delayed) cholecystectomy at increasing time points.

**Methods:**

Hospitalisation and death data were linked for individuals aged ≥ 50 years admitted to hospital in New South Wales, Australia with mild GSP between 2008–2018. Primary outcome was interval cholecystectomy timing. Secondary outcomes included mortality, emergency readmission for gallstone-related disease (GSRD) (28 and 180-day), and length of stay (LOS) (index admission and total six-month GSRD).

**Results:**

3,003 patients underwent interval cholecystectomy: 861 (28.6%) at 1–30, 1,221 (40.7%) at 31–90 and 921 (30.7%) at 91–365 days from index admission. There was no difference in 365-day mortality between groups. Longer delay to cholecystectomy was associated with increased 180-day emergency GSRD readmission (17.5% vs 15.8% vs 19.9%, *p* < 0.001) and total six-month LOS (5.9 vs 8.4 vs 8.3, *p* < 0.001). Endoscopic retrograde cholangiopancreatography (ERCP) was increasingly required with cholecystectomy delay (14.5% vs 16.9% vs 20.4%, *p* < 0.001), as were open cholecystectomy procedures (4.8% vs 7.6% vs 11.3%, *p* < 0.001). Extended delay was associated with patients of lower socioeconomic status, regional/rural backgrounds or who presented to a low volume or non-tertiary hospital (*p* < 0.001).

**Conclusion:**

Delay to interval cholecystectomy results in increased rates of emergency readmission, overall LOS, risks of conversion to open surgery and need for ERCP. Index admission cholecystectomy is still recommended, however when not possible, interval cholecystectomy should be performed within 30 days to minimise patient risk and healthcare burden.

## Background

Gallstone disease is a leading cause of acute pancreatitis comprising 40–70% of all cases [1]. Laparoscopic cholecystectomy is recommended for patients presenting with gallstone pancreatitis (GSP) to prevent recurrence [2]. Optimal timing of cholecystectomy is dependent on episode severity as classified by the Revised Atlanta Criteria [1, 2]. In cases of mild GSP, characterised by the absence of organ failure and local or systemic complication, international guidelines recommend laparoscopic cholecystectomy be performed during the same (index) admission [2–5]. Delay in cholecystectomy beyond index admission is associated with increased risk of recurrent gallstone-related disease (GSRD) including pancreatitis, cholecystitis, and cholangitis [5–7]. A systematic review by Van Baal et al [8] identified an 18% greater GSRD readmission rate within 90 days of index admission for mild GSP for those in whom cholecystectomy was delayed.

Index cholecystectomy for mild GSP is not always feasible [5], this may be due to patient, service, or surgeon factors. These include provision and access to emergency surgical services [9], surgeon availability and to allow for medical optimisation of patients or for medications such as antithrombotic agents to be withheld. This is seen more commonly in the older population, with poor adherence to guideline care demonstrated internationally [10] despite evidence supporting safe laparoscopic surgery in older people [11–14]. A recent ten year population-based study revealed a 38% adherence to index cholecystectomy in a cohort aged 50 and above in New South Wales, Australia [5]. Compared to index cholecystectomy, patients undergoing interval cholecystectomy were at greater risk of emergency readmission secondary to GSRD and open cholecystectomy procedures [5]. Investigation into the timing of interval cholecystectomy may provide further insight into the variation in healthcare provision and impact on outcomes. This population-based retrospective cohort study aims to 1) quantify timing of interval cholecystectomy and 2) compare mortality, emergency readmission and length of stay (LOS) associated with increasing delay in interval cholecystectomy for mild GSP in individuals aged ≥ 50 years across all hospitals in New South Wales (NSW), Australia over a 10-year period.

## Methods

### Study design

The study cohort was identified from the Surgical Care of Older People (SCOPE) cohort; a linked dataset comprising linked hospital (Admitted Patient Data Collection (APDC)) and death (Register of Births, Deaths, and Marriages (RBDM) data for all adults aged ≥ 50 years admitted to all NSW hospitals under a surgical specialty between 1 January 2007 and 1 July 2019. The APDC is a census of all inpatient services provided in NSW public, private and day-stay facilities and contains information on patients’ demographics, diagnoses, and procedures, coded using the International Classification of Diseases and Related Health Problems, Tenth Revision, Australian Modification (ICD-10-AM) and Australian Classification of Health Interventions (ACHI) [15, 16]. Ethics approval for this study was obtained from the NSW Population and Health Services Research Ethics Committee (2018HRE0201). Study design, data abstraction and findings were reported in accordance with The Strengthening the Reporting of Observational Studies in Epidemiology (STROBE) guidelines [17].

### Case inclusion criteria

We identified all admitted patients aged ≥ 50 years with a primary diagnosis of acute GSP from 1 July 2008 to 30 June 2018, who underwent cholecystectomy within 12 months of index admission. Index admission with acute GSP was identified using the primary diagnosis ICD-10-AM codes K85.1 – ‘biliary acute pancreatitis’ and K85.9—‘acute pancreatitis, unspecified’ [15]. Interval admission cholecystectomy was defined as cholecystectomy delayed to a subsequent admission at least 24 h after index pancreatitis admission. An eighteen-month exclusion period prior to 1 July 2008 was implemented to preclude recurrent cases, ensuring only first presentations of acute GSP were captured. Patients admitted after 30 June 2018 were excluded to allow a one-year follow-up for all included patients.

Cholecystectomy was defined using the ACHI procedure codes: 30445–00, 30448–00, 30449–00, 30443–00, 30454–01, 30455, 30446–00 and 90343 [16]. Patients with surrogate markers of severe GSP such as major pancreatic dissection (30577–00), cholecystostomy (30375–05) and intensive care unit (ICU) admission during stay, were excluded. Patients with records of multiple cholecystectomies were excluded as they were unlikely to represent mild cases, or were coded in error.

### Creation of study variables and outcomes

Consistent with standardised urgency categorisation for elective surgery in NSW, Australia, interval cholecystectomy was categorised into groups based on time between index admission and cholecystectomy: 1–30, 31–90 and 91–365 days. The primary outcome measure was timing of interval cholecystectomy. Secondary outcomes were: 365-day all-cause mortality, 28 and 180-day emergency GSRD readmission (from discharge date of index admission), index admission LOS and total six-month LOS secondary to GSRD. GSRD referred to any cholelithiasis-derived pathology and was defined using ICD-10-AM codes: cholelithiasis/choledocholithiasis (K80.2, K80.5, K80.8), cholecystitis (K80.0, K80.1, K80.4, K81.0, K81.8, K81.9), cholangitis (K80.3, K83.0), pancreatitis (K85.1, K85.8, K85.9), obstruction, perforation or fistulisation of the biliary tract (K83.1, K83.2, K83.3), spasm of sphincter of Oddi (K83.4), and unspecified disease of the gallbladder or biliary tract (K82, K83.9) [15]. LOS of each ‘episode of care’ in hospital was defined as the difference in days between hospital discharge date and admission date**.** Hospitalisations that consisted of multiple contiguous episodes of care, where the separation code was a transfer to another hospital or type change transfer, were considered as one hospital stay and included in the LOS calculation. Reflective of potentially atypical care practices, rare complications, or coding errors, records with LOS greater than 3 standard deviations above the mean were excluded [18].

### Demographic characteristics

Patient demographic characteristics examined included: sex, age, Charlson Comorbidity Index (CCI), private health insurance status recorded at time of admission, Socio-Economic Index for Areas-Index of Relative Socio-economic Disadvantage (SEIFA-IRDS) and Accessibility/Remoteness Index of Australia (ARIA +). The 17 comorbidities contributing to the CCI were identified using the Quan ICD-10-AM coding algorithm [19], which has been shown to provide good prediction for 30 and 365-day mortality in the older surgical population [20]. A one-year lookback period was used to optimise identification of comorbidities [21]. Comorbidity was categorised into three groups (0, 1–2 and ≥ 3 comorbidities). CCI ≥ 3 was considered multimorbid. SEIFA-IRSD enabled ranking of geographic areas of residence into quintiles (1 = most disadvantaged, 5 = most advantaged) based on socioeconomic status [22]. ARIA + provided classification of remoteness into major city, inner regional, outer regional, remote and very remote based on physical distance from nearest service centres [23].

### Hospitalisation characteristics

Hospitalisation characteristics included: type of cholecystectomy performed (laparoscopic (30445), laparoscopic with bile duct exploration (30448–00 and 30449–00), open including conversion (30443, 30454–01 and 30446–00)), associated procedures/complications (cholangiogram (30439), endoscopic retrograde cholangiopancreatography (ERCP) with sphincterotomy (30485), biliary bypass (31472), repair of bile duct injury (30472)), surgical centre volume, defined by average number of cholecystectomies performed each year (low volume < 52, medium volume 52–156 and high volume > 156 cases/year) and hospital type of index admission and surgery (public tertiary, public non-tertiary and private).

### Statistical analysis

Statistical analysis was undertaken using SAS Enterprise Guide v7.1 (SAS Institute Inc, Cary NC, USA). Descriptive statistics were used to characterise cohort demographics and assess differences in secondary outcomes between 1–30, 31–90 and 91–365-day interval cholecystectomies. Cochran-Armitage test for trend (1-sided) was used to test for increased proportions across the increasing time periods, and ANOVAs to compare differences in means for continuous variables (age and LOS). Post-hoc tests using Tukey’s p-value adjustment were used to determine where the between group difference occurred. Univariate binomial regression was used to test the association of individual demographic and hospital characteristics on delayed interval cholecystectomies (defined as > 30 days between index admission and cholecystectomy) and reported as relative risk (RR) with 95% confidence intervals.

## Results

A total of 3,003 patients were identified as meeting the inclusion criteria of age ≥ 50 years, admitted to a NSW hospital with acute GSP who received interval cholecystectomy within a one year period. Of these, 861 (28.6%) occurred 1–30 days from index admission, 1,221 (40.7%) occurred 31–90 days from index admission and 921 (30.7%) occurred 91–365 days from index admission. Median time from index admission to interval cholecystectomy was 56 (IQR 27–107) days.

Demographic and hospitalisation characteristics of 1–30-, 31–90- and 91–365 day interval cholecystectomies are shown in Table 1. Increasing delay from index admission was associated with a higher proportion of patients that were male (49.8% vs 51.7% vs 53.9%, z = 2.2, *p* = 0.015), older (67.0 (± 10.4) vs 68.9 (± 10.6) vs 68.4 (± 10.3) years, *p* < 0.001) and multimorbid (20.7% vs 25.1% vs 28.7%, z = -3.8, *p* < 0.001). Likewise, the proportion of patients in the most socioeconomic disadvantaged quintile (23.2% vs 28.3% vs 32.9%, z = -4.5, n = 2,951, *p* < 0.001), with no private health insurance (48.9% vs 60.4% vs 72.7%, z = 9.9, *n* = 2,746, *p* < 0.001) and residing in outer regional/remote/very remote settings (5.7% vs 6.2% vs 10.7%, z = -4.0, *n* = 2,953, *p* < 0.001) increased with operative delay.

**Table 1:** Cohort characteristics at index admission, by timing of interval cholecystectomy, patients aged ≥ 50 years admitted with mild gallstone pancreatitis, NSW, 2008–2018

Characteristic	Cholecystectomy 1–30 days	Cholecystectomy 31–90 days	Cholecystectomy 91–365 days	Statistic
Patients	861	1,221	921	
Demographic Characteristics				
Sex				*p* = 0.015
Male	429 (49.8%)	631 (51.7%)	506 (54.9%)	
Age				
Mean years (SD)	67.0 (10.4)	68.9 (10.6)	68.4 (10.3)	*p* < 0.001
Charlson Comorbidity Index (CCI)				
Number of comorbidities				*p* < 0.001
0	398 (46.2%)	448 (36.7%)	293 (31.8%)	
1–2	285 (33.1%)	467 (38.3%)	364 (39.5%)	
≥3	178 (20.7%)	306 (25.1%)	264 (28.7%)	
Private health insurance status on admission^†^				*p* < 0.001
Yes	402 (51.1%)	435 (39.6%)	235 (27.3%)	
No	385 (48.9%)	664 (60.4%)	625 (72.7%)	
SES based on residence (SEIFA-IRSD)—Quintiles^‡^				*p* < 0.001
1^st^ (lowest)	194 (23.2%)	341 (28.3%)	299 (32.9%)	
2nd	206 (24.6%)	303 (25.2%)	257 (28.2%)	
3rd	162 (19.4%)	239 (19.9%)	158 (17.3%)	
4th	122 (14.6%)	160 (13.3%)	101 (11.1%)	
5th	153 (18.3%)	161 (13.4%)	96 (10.4%)	
Accessibility/Remoteness Index of Australia (ARIA +)^§^				*p* < 0.001
Major city	581 (69.2%)	841 (69.8%)	581 (63.9%)	
Inner regional	210 (25.0%)	288 (23.9%)	232 (25.5%)	
Outer regional/remote/very remote	48 (5.7%)	75 (6.2%)	97 (10.7%)	
Hospitalisation Characteristics				
Type of Surgery				*p* < 0.001
Laparoscopic cholecystectomy	785 (91.2%)	1,080 (88.4%)	801 (87.0%)	
Laparoscopic cholecystectomy with BDE	35 (4.1%)	48 (3.9%)	16 (1.7%)	
Open cholecystectomy procedures	41 (4.8%)	93 (7.6%)	104 (11.3%)	
Other procedures				
Cholangiogram	768 (89.2%)	985 (80.7%)	726 (78.8%)	*p* < 0.001
ERCP Sphincterotomy	125 (14.5%)	206 (16.9)	188 (20.4)	*p* < 0.001
Biliary bypass	¶	¶	¶	*p* < 0.001
Repair of bile duct injury	¶	¶	¶	*p* < 0.001
Surgical Centre volume				*p* < 0.001
Low volume [< 52/year]	92 (10.7%)	169 (13.8%)	162 (17.6%)	
Medium volume [52–156/year]	468 (54.4%)	651 (53.3%)	462 (50.2%)	
High volume [> 156/year]	301 (35.0%)	401 (32.8%)	297 (32.2%)	
Hospital type, of admission				*p* < 0.001
Public tertiary referral	356 (41.4%)	449 (36.8%)	323 (35.1%)	
Public non-tertiary	486 (56.5%)	751 (61.5%)	584 (63.4%)	
Private	19 (2.2%)	21 (1.7%)	14 (1.5%)	
Hospital type, of surgery				*p* < 0.001
Public tertiary referral	200 (23.2%)	318 (26.0%)	323 (35.1%)	
Public non-tertiary	301 (35.0%)	481 (39.3%)	406 (44.1%)	
Private	360 (41.8%)	422 (34.5%)	192 (20.9%)	

^†^ 257 (8.6%) patients excluded as private insurance not assigned

^‡^ 52 (1.7%) patients excluded as SEIFA-IRDS not assigned

^§^ 50 (1.7%) patients excluded as ARIA + not assigned

^¶^ cell sizes ≤ 5 supressed

Laparoscopic cholecystectomy was performed in 92.1% of cases with proportionally less cholangiogram use with increasing delay in interval cholecystectomy (89.2% vs 80.7% vs 78.8%, z = 5.7, *n* = 3,003, *p* < 0.001). There was a greater proportion of open cholecystectomy procedures (4.8% vs 7.6% vs 11.3%, z = 5.1, *n* = 3,003, *p* < 0.001) and ERCP with sphincterotomy requirement (14.5% vs 16.9% vs 20.4%, z = -3.3, *n* = 3,003, *p* < 0.001) with increasing operative delay. Patients initially admitted to low surgical volume hospitals (10.7% vs 13.8% vs 17.6%, z = -4.2, *n* = 3,003, *p* < 0.001) and public non-tertiary centres (56.5% vs 61.5% vs 63.4%, z = -3.0, *n* = 3,003, *p* = 0.001) were proportionally more likely to experience increased delay in cholecystectomy. Additionally, patients who underwent subsequent cholecystectomy in a private hospital were proportionally more likely to receive earlier surgery (41.8% vs 34.5% vs 20.9%, z = 9.4, *n* = 2,994, *p* < 0.001).

There was no significant difference in 365-day mortality following index presentation with GSP between the interval cholecystectomy groups (Table 2). Twenty eight day readmission was highest in those undergoing surgery within 30 days post index admission (15.7%), whilst 180 day readmission was highest in those with the longest delay to surgery (19.9%).

**Table 2:** Outcomes, by timing of interval cholecystectomy, patients aged ≥ 50 years admitted with mild gallstone pancreatitis, NSW, 2008–2018

Characteristic	Cholecystectomy 1–30 days	Cholecystectomy 31–90 days	Cholecystectomy 91–365 days	Statistic
Patients	861	1,221	921	
Outcomes				
Mortality				
365 days	15 (1.7)	26 (2.1)	12 (1.3)	*p* = 0.56
Re-admissions				
28-day GSRD *emergency* readmission	135 (15.7)	63 (5.2)	48 (5.1)	*p* < 0.001
180-day GSRD *emergency* readmission	151 (17.5)	193 (15.8)	183 (19.9)	*p* < 0.001
Number of readmissions				
1	131 (15.2)	168 (13.8)	152 (16.5)	
2 +	20 (2.3)	25 (2.0)	31 (3.3)	
Length of Stay (LOS in days)				
LOS for index admission, mean days (SD)^††^	4.3 (2.7)*, **	5.6 (4.1)*	5.8 (4.4)**	*p* < 0.001
6-month LOS secondary to GSRD (Excluding index admission), mean days (SD)^‡‡^	2.9 (3.4)*	3.2 (4.2)	3.8 (4.7)*	*p* < 0.001
TOTAL 6-month LOS (Index admission + readmissions), mean days (SD)^&^	6.9 (4.6)*, **	8.4 (5.8)*	8.3 (6.7)**	*p* < 0.001

SD standard deviation

IQR interquartile range

^††^ 73 (2.4%) patients > 3 standard deviations above the mean excluded

^‡‡^ 41 (1.4%) patients > 3 standard deviations above the mean excluded & 81 (2.7%) patients > 3 standard deviations above the mean excluded

*, ** significant difference between groups based on post-hoc tests

Mean LOS of index admission was significantly longer with deferral in cholecystectomy beyond 30 days (4.3 (± 2.7) vs 5.6 (± 4.1) and 5.8 (± 4.4), *p* < 0.001). Similarly, six-month mean LOS secondary to emergency readmissions was greater with increasing cholecystectomy delay (2.9 (± 3.4) vs 3.2 (± 4.2) vs 3.8 (± 4.7) days (p < 0.001). Cumulative LOS of index admission and emergency readmissions at six months was significantly shorter following 1–30 day cholecystectomy compared to cholecystectomy performed at 31–90 and 91–365 days (6.9 (± 4.6) vs 8.4 (± 5.8) and 8.3 (± 6.7) days, *p* < 0.001).

Univariate binomial regression analysis (Table 3) identified demographic and hospitalisation risk factors for delayed cholecystectomy beyond 30 days of discharge. Age (RR 1.00; 0.95%CI 1.00–1.01), not having private health insurance (RR 1.23; 95%CI 1.17–1.30) and being co-morbid (1–2: RR 1.15; 0.95%CI 1.08–1.21, 3: 1.17; 0.95%CI 1.10–1.24) were individual risk factors for delayed interval cholecystectomy. People of the lowest socioeconomic quintile (RR 1.23; 0.95%CI 1.28–1.33) and residing in outer regional/remote/very remote areas (RR 1.10; 0.95%CI 1.1.02–1.19) were associated with 23% and 10% increased risk of having a delayed cholecystectomy over 30 days from discharge, respectively. People whose index admission were to low volume (RR 1.12; -0.95%CI 1.05–1.19) or non-tertiary/private hospitals (RR 1.07; 0.95%CI 1.01–1.12) were 12% and 7% more likely to undergo delayed interval cholecystectomy.

**Table 3:** Demographic and hospital factors associated with delayed interval cholecystectomy (> 30 days), patients aged ≥ 50 years admitted with mild gallstone pancreatitis, NSW, 2008–2018

Characteristic	Unadjusted RR (95% CIs)	*P* value
Sex		
Female	1.00	
Male	1.04 (0.99–1.09)	0.1072
Age, years	1.00 (1.00–1.01)	** < 0.0001**
CCI		
0	1.00	
1–2	1.15 (1.08–1.21)	** < 0.0001**
≥ 3	1.17 (1.10–1.24)	** < 0.0001**
Private health insurance		
Yes	1.00	
No	1.23 (1.17–1.30)	** < 0.0001**
SES quintile		
5^th^ (highest)	1.00	
4^th^	1.09 (0.98–1.21)	0.1006
3^rd^	1.13 (1.04–1.24)	**0.0070**
2^nd^	1.17 (1.07–1.27)	**0.0004**
1^st^ (lowest)	1.23 (1.28–1.33)	** < 0.0001**
ARIA +		
Major city	1.00	
Inner regional	1.00 (0.95–1.06)	0.9026
Outer regional/remote/very remote	1.10 (1.02–1.19)	**0.0120**
Hospital type (admission hospital)		
Public tertiary	1.00	
Non tertiary/private	1.07 (1.01–1.12)	**0.0078**
Surgical centre volume		
High [> 156/year]	1.00	
Medium [52–156/year]	1.01 (0.97–1.06)	0.7754
Low [< 52/year]	1.12 (1.05–1.19)	**0.0006**

Bold entries represent those of statistical significance (*p* < 0.05)

## Discussion

This population-based study provides insight into the management of people ≥ 50 years of age with mild GSP, in NSW, over 10 years to 2018 and the impact of delayed surgical intervention.

These data give an indication of the ‘current state of play’ in NSW hospitals in respect to cholecystectomy for this disease. Index cholecystectomy is regarded ‘best practice’ for mild GSP. It is recognised that this is not always feasible for a variety of reasons. However the results from this study show that increasing delay is associated with negative outcomes for both patients and the health system. The increasing utilisation of acute surgical units and protected emergency operating times has been recommended and implemented in larger centres [9] but may be impossible to implement in smaller centres due to funding and staffing constraints. Where surgery on index admission is not possible, this study provides data to support a recommendation for admission within 30 days as opposed to 90 and 365 days.

In this population, interval cholecystectomy was performed with considerable delay; 71.3% of interval cholecystectomies were deferred beyond 30 days of index admission and on average performed at 56 days. Despite no difference in mortality, increasing delay in cholecystectomy was significantly associated with recurrent emergency readmission, greater LOS and unnecessarily complex or additional procedures. 91–365 day cholecystectomy posed the greatest overall risk of emergency GSRD readmission. This was concordant with the significantly greater requirement for ERCP associated with cholecystectomy delay beyond 30 days, likely characterising the recurrence of biliary obstruction, cholangitis or the finding of choledocholithiasis.

High emergency readmission at 28 days was observed in the 1–30 day interval cholecystectomy group. We hypothesise this may reflect those with recurrent symptoms requiring readmission who, in most cases, then undergo a second admission emergency cholecystectomy; rather than elective. This is consistent with the average time to GSRD readmission of within 28 days of discharge in previous studies, and the comparatively low 28-day emergency GSRD readmission rates found in the 31–90 and 91–365 day cholecystectomy groups [6, 24]. This highlights a cohort of patients who would have clearly benefited from index or early interval cholecystectomy.

Older patients with GSP are at higher risk of complicated biliary tract disease necessitating open cholecystectomy procedures [25, 26]. Classical surgical teaching dictates delaying cholecystectomy to allow resolution of inflammation and improve chances of a laparoscopic procedure. However, delay in cholecystectomy increases the risk of both inability to perform a cholangiogram and conversion to an open procedure, as shown in this population and described in previous series of the older cohort [10, 27]. Furthermore, delay results in a greater ERCP requirement, which is associated with a more challenging subsequent cholecystectomy with longer operative times and risks of open conversion [28–30]. Collectively, this may result from recurrent acute/chronic inflammation, bacterial colonisation and scarring of the hepatoduodenal ligament, intra-abdominal adhesions or patient factors not definable by this study [28].

Overall, interval cholecystectomy performed within 30 days of discharge was superior, and on average at least a 1.3 day shorter index admission and 1.4 day shorter total LOS at six months than further delayed counterparts. This increase in LOS likely reflects the culmination of increased emergency GSRD readmissions, ERCPs and open cholecystectomy procedures associated with increasing operative delay.

Our findings highlight clear variation in the scheduling of interval cholecystectomy. Scheduling cholecystectomy within 30 days results in a decreased total six-month LOS and reduces emergency GSRD readmissions, the need for ERCP with sphincterotomy and conversion to open procedures. Accordingly, interval cholecystectomy performed as early as possible and within 30 days of index admission, may provide the best comparable outcomes to gold standard care with index cholecystectomy [5]. Given the increased patient risk and health service burden associated with delay, interval cholecystectomy for mild GSP needs to be prioritised through national standardised recommendation for admission as urgent (Category 1), and ideally no later than 30 days from index admission.

Demographic and hospitalisation characteristics identified in this study represent risk factors for excessive delay in cholecystectomy and warrant further exploration. Lower socioeconomic status, regional and remote residence and admission to hospitals of low surgical volume and non-tertiary status were significantly associated with delay in interval cholecystectomy. Whilst this may in part reflect surgeon perception and availability, it highlights inequitable provision of health care. Regional centres carry a significant burden of emergency general surgery admissions [31] and insufficient access to emergency operating time acts to promote deferral of definitive management and further exacerbate elective surgery wait times. Furthermore, limited surgical outpatient services in rural and regional settings can exacerbate delay to outpatient review and recommendation for admission for elective cholecystectomy. This situation can foreseeably precipitate cases of unacceptable delays in cholecystectomy and typifies the known disparity in healthcare across Australia. It highlights the importance of adherence to the NSW Emergency Surgery Guidelines produced by the NSW Agency for Clinical Improvement and principles in matching dedicated emergency operating time to predicted local emergency case mix [9]. This model of care has been further validated in the Australian rural setting; the area for which our findings would suggest it is most needed [31]. These findings precede the COVID-19 pandemic which has led to a substantial increase in time to surgery for elective procedures [32]. Although private health insurance may provide an alternate avenue for early access to definitive management, this should not be seen as a replacement for early access through the public healthcare system. Additionally, whilst delay in favour of optimisation of pre-existing comorbidities is reasonable assuming that optimisation is achievable, this should be limited to within 30 days of discharge from index admission where possible given the increased risks associated with further delay.

The strengths of this study include its size and population-based design which allows exploration of important patient and system level confounding factors. There was no loss to follow up based on study design, and readmissions, excluding those interstate, were accurately accounted for as the administrative hospital data includes all presentations to all NSW hospitals. There are however inherent limitations to administrative data. The databases supporting this study design are not established primarily for research and reliance is placed on accurate diagnosis and procedural coding. Values for private health insurance, SEIFA-IRDS, ARIA + and urgency of admission were not assigned in some individuals and therefore excluded from respective characteristic analyses. Furthermore, direct measures of acute pancreatitis severity such as end organ failure and local or systemic complications stipulated in the Revised Atlanta Classification, were not available. Proxy variables representative of moderately severe/severe cases of GSP such as major pancreatic resection, cholecystostomy and ICU admission during index admission were employed to address this. Complications such as bile duct injury and bile duct reconstruction, that potentially necessitate ICU admission are at risk of exclusion and underrepresentation in this data set. Differentiation of ICU admission into planned or emergency and pre- or post-cholecystectomy may have provided further insight into pancreatitis severity, however these data were not available.

## Conclusion

In this population aged 50 and above, only one third of patients underwent interval cholecystectomy within 30 days of index admission. Delay in surgery beyond 30 days is associated with increasing risk of emergency readmission, ERCP and conversion to open cholecystectomy. Index cholecystectomy remains the treatment of choice, but when this is not possible, interval cholecystectomy should be recommended for admission within 30 days of discharge from hospital to minimise these risks and prevent undue health service burden.

## Data Availability

The data that support the findings of this study are available from NSW Ministry of Health and NSW Registry of Births, Deaths and Marriages but restrictions apply to the availability of these data, which were used under license for the current study, and so are not publicly available. Data may be available from the authors upon reasonable request and with permission of the NSW Ministry of Health / RBDM.

## Abbreviations

*GSP*: Gallstone pancreatitis
*GSRD*: Gallstone-related disease
*ERCP*: Endoscopic retrograde cholangiopancreatography
*LOS*: Length of stay
*NSW*: New South Wales
*SCOPE*: Surgical Care of Older People
*APDC*: Admitted Patient Data Collection
*RBDM*: Register of Births, Deaths, and Marriages
*ICD-10-AM*: International Classification of Diseases and Related Health Problems, Tenth Revision, Australian Modification
*ACHI*: Australian Classification of Health Interventions
*STROBE*: Strengthening the Reporting of Observational Studies in Epidemiology
*ICU*: intensive care unit
*CCI*: Charlson Comorbidity Index
*SEIFA-IRDS*: Socio-Economic Index for Areas-Index of Relative Socio-economic Disadvantage
*ARIA +*: Accessibility/Remoteness Index of Australia
*SD*: Standard deviation
*IQR*: Interquartile range
